# How experience makes a difference: practitioners’ views on the use of deferred consent in paediatric and neonatal emergency care trials

**DOI:** 10.1186/1472-6939-14-45

**Published:** 2013-11-06

**Authors:** Kerry Woolfall, Lucy Frith, Carrol Gamble, Bridget Young

**Affiliations:** 1 Department of Psychological Sciences, University of Liverpool, Liverpool, UK; 2 Department of Health Service Research, University of Liverpool, Liverpool, UK; 3 Department of Biostatistics, University of Liverpool, Liverpool, UK

## Abstract

**Background:**

In 2008 UK legislation was amended to enable the use of deferred consent for paediatric emergency care (EC) trials in recognition of the practical and ethical difficulties of obtaining prospective consent in an emergency situation. However, ambiguity about how to make deferred consent acceptable to parents, children and practitioners remains. In particular, little is known about practitioners’ views and experiences of seeking deferred consent in this setting.

**Methods:**

As part of a wider study investigating consent methods in paediatric emergency care trials (called CONNECT), a 20 item online questionnaire was sent by email inviting practitioners (doctors and nurses) who were involved in talking with families about children’s and young people’s (aged 0–16 years) participation in UK EC trials. To ensure those with and without experience of deferred consent were included, practitioners were sampled using a combination of purposive and snowball sampling methods. Simple descriptive statistics were used to analyse the quantitative data, whilst the constant comparative method was used to analyse qualitative data. Elements of a symbiotic empirical ethics approach was used to integrate empirical evidence and bioethical literature to explore the data and draw practice orientated conclusions.

**Results:**

Views on deferred consent differed depending upon whether or not practitioners were experienced in this consent method. Practitioners who had no experience of deferred consent reported negative perceptions of this consent method; these practitioners were concerned about the impact that deferred consent would have upon the parent-practitioner relationship. In contrast, practitioners experienced in deferred consent described how families had been receptive to the consent method, if conducted sensitively and in a time appropriate manner. Experienced practitioners also described how deferred consent had improved recruitment, parental decision-making capacity and parent-practitioner relationships in the emergency care setting.

**Conclusions:**

The views of practitioners with first-hand experience of deferred consent should be considered in the design and ethical review of future paediatric EC trials; the design and ethical review of such trials should not solely be informed by the beliefs of those without experience of using deferred consent. Further research involving parents and children is required to inform practitioner training and normative guidance on the use and appropriateness of deferred consent in emergency settings.

## Background

Clinical trials of interventions to save the lives of critically ill children are vital. However, recruitment to clinical trials in the emergency care (EC) setting is fraught with practical and ethical dilemmas. Children cannot legally provide consent for their own participation in a clinical trial; instead consent is sought from a parent or legal guardian in addition to child assent [1,2]. Prior written consent may not be logistically possible as parents are not always present when a child enters hospital, or a newly delivered mother maybe heavily sedated preventing prior consent for a newborn to enter a study. In circumstances where parents are present the therapeutic window for successful intervention may be too short to seek informed consent [3], or parents may lack the capacity to provide adequately informed consent when their child is critically ill [4]. The EC setting is time constrained and intensely emotional, posing ethical difficulties for practitioners (doctors and nurses) recruiting to clinical trials in ensuring that parental consent is informed, participation is voluntary and the recruitment process adheres to bioethical principles that govern research ethics and underpin good professional practice, such as respect for autonomy, justice, nonmaleficence and beneficence [5,6].

Empirical research has indicated that practitioners are anxious about approaching parents for consent to clinical trials even outside of the emergency setting [7]. Seeking informed consent from parents in the emergency setting has been regarded as needlessly cruel, compromising individual autonomy and violating the principle of beneficence [3,6-8]. The ethical validity and practical feasibility of obtaining informed consent in this setting has also been questioned [9]. To address these complexities, one proposal is that an initial assessment is undertaken to establish parents’ competence to understand trial information and to evaluate capacity to provide informed consent [8,10]. Consent prior to a child’s entry into a trial would only be sought if a parent was deemed to be competent. This proposal includes a formal assessment stage including the use of outcome assessment tools, which are not commonly used in conventional approaches to consent [10]. Alternatively a staged (continuous consent) approach has been proposed, which involves brief information provision prior to randomisation followed by retrospective consent [11]. However, parental capacity to process brief information provision and complete an assessment in this setting may be questioned. Even brief information provision could inappropriately burden parents when they are highly stressed. It could also delay the treatment of a critically ill child, particularly if parents have questions about the trial. Waived and deferred consent methods, whereby research can proceed without prospective consent, have been proposed as potential solutions to such difficulties [9].

Research without prospective consent prevents the need to provide parents with information and burden them with a request for decision making at a time when their child’s condition is in the most critical stage. Some have argued that deferring or waiving consent enables vital research to proceed that benefits society as a whole and avoids potentially harmful delays to the individual in receiving the medical intervention [12,13]. However, conducting research without prior consent has been subject to much debate. It poses a key ethical dilemma as this approach prevents personal choice and therefore erodes individual autonomy [14-16]. Consent for paediatric clinical trials is sought from parents rather than children [17] and in such trials it could be argued that deferred consent is a further detraction from the patient autonomy principle. As children (and parents in trials that use deferred consent) have no control over whether interventions are administered deferred consent may also compromise rights based justice and the requirement to act in a fair manner [18]. Equity may also be compromised if vulnerable groups, such as bereaved parents, find it particularly difficult to understand trial information and make a consent decision due to situational incapacity at the point when deferred consent is sought [19]. However, others have argued that because practitioners prescribe untested or unlicensed treatments on a daily basis, distributive justice should be the overriding principle to ensure that all children benefit from evidence based treatments in the future [13,18]. In United States of America and some parts of Europe, research without consent is permitted in emergency settings where the research is perceived to be of minimal risk to participants. Practitioners are required to consult with representatives of the patient community and post public notices of the study protocol, risks, benefits and results. How such consultation is implemented appears to vary across studies. Some approaches are similar to deferred consent [20,21], as practitioners often (but not always) approach families with information at a later stage when the situation is no longer critical and provisions are made so that patients (or parents of children) can ‘opt out’ of the trial if they wish [22,23]. Clinical research is governed by European Community Directives (2001/20/EC and 2005/28/EC) which set the legal framework [24] for valid informed consent as the cornerstone of experimental research involving human beings. However, these Directives made no provision for consent in emergency situations, thus creating a barrier to research in this setting [25].. However, in 2006, UK legislation was amended to incorporate deferred consent in emergency situations for adults [26], and this extended to children in 2008 [27].

Deferred consent was first described by Fost and Robinson (1980) and involves enrolling a patient to a trial then seeking consent (from patients if they have capacity or alternatively a surrogate decision maker) after enrolment and administration of trial treatments in order for the patient to continue in the trial [28]. Community consultation is not required and consent is sought for permission to use data that have already been collected. It has been argued deferred consent addresses many of the difficulties which compromise autonomous decision making in the emergency setting, such as constraints on time and parental capacity [11]. However, some have argued that deferred consent is ethically unsound and represents a dishonest attempt to justify recruitment without consent [11,29]. It has been proposed that deferred consent compromises the core value of informed consent and that it is the role of ethicists to ensure that informed decision making is defended [29]. A critical issue as noted above is that by deferring consent seeking, a patient is being asked to consent for the use of data that has already been collected, or to consent to continue in the study, rather than for giving permission to be entered into the study in the first place, which is argued to be the key function of consent in medical research [30].

Despite these criticisms, deferred consent can be used in UK clinical trials, although ambiguity about how to make it practically and ethically acceptable to parents, children and practitioners remains. In particular, little is known about practitioners’ views and experiences of seeking deferred consent in this setting [4,15]. Do they have anxieties about deferring consent? What are their perceptions of this method? And what are their experiences of how parents react to finding out their child has been entered into a trial? A key concern is that if the decision to provide consent is associated with the outcome then the trial results will be biased. And what happens if a child dies before consent is sought? Excluding deceased patients could lead to bias [31]. However, is it ethically appropriate to approach bereaved parents? Empirical research is required to help address these and other key ethical questions to inform future clinical practice in this setting, such as: does deferred consent reduce individual autonomy? Does it compromise parental trust in the medical profession and negatively impact upon immediate parent-practitioner relationship? Or can deferred consent promote beneficence by supporting situational capacity to provide informed consent at a later stage when children are critically ill? [32].

To begin to answer these questions, this paper draws on insights from empirical data from an online survey on the views and experiences of clinical trial practitioners working in paediatric and neonatal emergency care settings. We purposively sampled practitioners to include those with experience of using deferred consent and those who had not previously used this method of consenting.

## Methods

### Questionnaire

The findings we report here are part of a wider study, CONNECT (CONseNt methods in paediatric Emergency and urgent Care Trials). CONNECT is a mixed method study involving parents, children and practitioners from across the UK with experience of paediatric EC. It will generate new evidence to help improve how consent is sought for paediatric clinical trials in emergency situations. Findings presented in this paper are derived from the first stage of CONNECT; further findings will be published in due course.

We designed a 20 item semi-structured questionnaire (See Additional file 1) to examine the experiences and attitudes of practitioners (doctors and nurses) involved in recruiting to clinical trials in the EC setting. We derived closed and open-ended questions from key issues identified in the EC and ethics literature on consent methods. Part A of the questionnaire contained questions on practitioners’ job description, experience and trial specific questions, including: the types of consent methods used in UK EC paediatric clinical trials (e.g. proxy, deferred, prospective, child assent); how the consent process is conducted and by whom; trial recruitment and consent materials (including the involvement of public and patient [PPI] representatives who are not trial participants but contribute to the research team). Part B contained questions to explore practitioners’ opinions on perceived barriers and facilitators to obtaining patient and proxy consent/assent; perceived impact of the trial recruitment and consent processes upon the patient-investigator relationship; and training and support needs. The questionnaire was designed so that participants could report on more than one trial by completing multiple Part As. Opinion and training questions in Part B were only answered once by each participant.

The questionnaire was reviewed by an expert advisory panel established to inform the design and conduct of CONNECT (n = 11) including: bio medical ethicists (n = 3); medical sociologists (n = 2); statisticians (n = 2); a health psychologist; a clinical trials manager; a patient and public involvement representative; and a paediatric intensive care research nurse. The questionnaire was firstly piloted in one North West Clinical Trials Unit (n = 7 questionnaires) with amendments made prior to dissemination. The study was approved by a UK National Health Service ethics committee (Northwest- Liverpool East Research Ethics Committee: 12/NW/0094).

### Survey procedure

The questionnaire was sent by email invitation to practitioners involved in talking with families about children’s and young people’s (aged 0–16 years) participation in UK EC trials. Each questionnaire was accompanied by a letter outlining the aims of the study and providing instructions on how to complete the online questionnaire. A participant information sheet was also attached which provided further details of the study, including confidentiality and data protection statements. Recruitment involved purposive sampling to help ensure both research nurses and doctors, as well as those with and without experience of deferred consent were included in the sample [33]. Snowball sampling was also used to access clinical trial recruiters whose details were not accessible to the researcher [9,34]. This involved distributing the questionnaire via an email link to all trial managers and clinical trial recruiters engaged in paediatric and neonatal emergency care trials through identified gatekeepers in each of the 42 UK clinical trial units and eight Medicines for Children Research Network (MCRN) Local Research Networks (LRN). In addition, KW contacted practitioners who were known to be recruiting to trials in this setting through (MCRN) Clinical Trials Unit (CTU) contacts. In order to access practitioners who had experience of deferred consent the questionnaire was also distributed to practitioners who were actively recruiting to the CATCH trial at the time that this survey was conducted. CATCH was a multi-centre UK trial that incorporated a deferred consent process to investigate interventions to reduce central venous catheter-associated infections in children [35]. For CONNECT, practitioners were asked to complete questionnaires for each paediatric emergency care trial they had been involved in since 2004. This date was used to identify consent methods used in trials conducted before and after the 2008 Medicines for Human Use (Clinical Trials) regulations, which incorporated deferred consent in paediatric emergency settings [24,27]. Snowball sampling helped boost the size and diversity of the sample; however this meant that response rates could not be calculated as the total population who received the questionnaire was not known [9,34]. Completion of the questionnaire was taken as an indication of consent. No identifiable personal data was collected.

### Analysis

Quantitative data from closed questions were analysed using simple descriptive statistics, chi-square test for trend and Fisher Exact test as appropriate. QSR NVivo 9 software was used to assist in the organisation and indexing of responses to open ended questions. Whilst analysis was informed by the constant comparison approach of grounded theory, the focus was modified to fit with the criterion of catalytic validity, whereby findings should be relevant to future research and practice [36,37]. Elements of a symbiotic empirical ethics approach was used to integrate empirical evidence and bioethical literature [38], explore the data and draw practice orientated conclusions. This included considering the data in relation to the circumstances which impact upon trial recruitment in this setting, and considering how key theories and principles could inform the analysis [38]. KW (a sociologist) led the analysis and development of coding framework with assistance from BY (a health psychologist) and LF (a bioethicist) to enable investigator triangulation [39]. The experience of practitioners (e.g. experienced or not experienced in deferred consent) is shown next to quotations presented in the results section.

## Results

### Participant characteristics

In total, 45 EC practitioners completed the online questionnaire. The sample comprised 16 (36%) consultant grade doctors and 29 (64%) research nurses. 39 (86.7%) worked in paediatric emergency care units and n = 6 (13.3%) worked in neonatal intensive care units.. Six (21%) of the research nurses were senior nurses. Twenty five (56%) practitioners had been involved in delivering a medical intervention in the context of any trial. Out of the 45 participants, the average number of trials they had recruited to was three (SD = 2.33), ranging from a minimum of one trial (n = 12, 27%) to a maximum of ten trials (n = 3, 7%). All participants provided at least one response to an open ended question. There were 222 open ended responses in total.

Thirty-eight practitioners completed one Part A (which contained trial specific questions) and seven completed more than one Part A relating to more than one trial. Three, two and two practitioners completed two, three, and four part As respectively for their involvement in multiple trials giving a total of 58 Part As completed (mean = 1.28 completed per participant, range 1–4). Opinion questions in Part B were only answered once by each participant (n = 45). Data were collected on 13 emergency and urgent care trials conducted since 2004. Five trials began between 2004 and 2008 (one in each year), whilst three started in 2009, four in 2010 and one in 2011. The majority (n = 9/13, 69%) began after UK legislation was amended in 2008 which enabled the use of deferred consent in paediatric emergency care trials [27].

In eight of the 13 (62%) trials, multiple types of consent methods were used, reflecting complex study designs (e.g. use of elective and emergency care arms within the same trial). Practitioners in all 13 trials sought prospective informed proxy consent from parents. Inclusion criterion specified child age ranges from birth up until (not including) 16 years of age. Deferred consent was used in the emergency arm of only one trial (CATCH), which (where appropriate) involved brief information provision prior to randomisation, followed by full information and deferred consent within an optimal 48 hours of randomisation. The TOBY trial [40] (which investigated whole body hypothermia for perinatal asphyxia) involved stages of consent (e.g. brief information followed by full information provision) although full informed consent was sought from all parents prior to enrolment and randomisation. Informed prospective assent was sought from children in four trials, all were urgent elective care trials (<16 years). Procedures for deferred assent from children were reported in two emergency care trials (CATCH and TOBY), whereby assent could be sought when a child’s condition had been stabilized and capacity to assent had returned. Nine practitioners from four trials where the protocol required brief written information to be provided to parents stated that they had deviated from the consent model outlined in the trial protocol. Open response comments at end of the questionnaire suggested that this was due to parents not being present in order to receive trial information.

Over half the sample (n = 27/45, 60%) of practitioners had experience of using deferred consent in an emergency or urgent care trial. As CATCH was the only trial identified by this survey as having used deferred consent, it is likely that the rest of the sample did not have the opportunity to use this consent method. Due to the sampling strategy used, a large proportion of part A questionnaires (n = 26/58, 45%) reported on the CATCH trial. The findings are presented under the key themes derived from the synthesis of qualitative and quantitative data analyses.

### Consent in the emergency context

Practitioners were asked to rate (from very well, to not at all well) how well they felt parents or family members understood the trial information provided in an emergency/urgent care situation. Just over half (n = 26/45, 58%) responded that parents understood the information well (n = 25/45, 56%), or very well (n = 1/45, 2%). Approximately a third (n = 14/45, 31%) were unsure. Few practitioners indicated that parents did not understand the information well (n = 4/45, 9%), or not at all well (n = 1/45, 1%). As shown in Table 1, there were no statistically significant differences between groups in their perceptions of perceived parental/family member understanding of trial information in an emergency situation by experience of deferred consent or job type (consultant or research nurse).

**Table 1:** **How well do you feel parents/family members understand the trial information given to them in an emergency/urgent care situation? (n** = **45)**

	**Very well**	**Well**	**Unsure**	**Not well**	**Not at all well**	**p-value**
Experienced in deferred consent	1 (4)	14 (52)	10 (37)	2 (7)	0 (0)	0.52
Not experienced in deferred consent	0 (0)	11 (61)	4 (22)	2 (11)	1 (6)	
Consultant	1 (6)	8 (50)	5 (31)	1 (6)	1 (6)	0.41
Research Nurse	0 (1)	17 (58)	9 (31)	3 (10)	0 (0)	

Figures are n (%), percentages are rounded to one decimal place.

Some practitioners questioned parents’ ability to provide informed consent in an emergency setting, particularly if consent was sought before prior to randomisation: *“I have been involved in many neonatal trials- many of which are emergency. We generally obtain consent before. It’s clear that people cannot be given FULLY informed consent because of the high emotion and urgency of the situation”* (P44, not experienced in deferred consent).

Some practitioners viewed recruitment in EC as being an additional burden for parents, who they regarded as being already highly anxious about their child, even when consent was deferred *“It* [clinical trial recruitment] *increases the parent/carer stress levels dramatically* (P20, experienced in deferred consent). Such comments were made by consultants and research nurses in the open comments section at the end of the questionnaire, suggesting that the impact of the emergency setting upon parents’ ability to provide consent was an important point which they wished to emphasise.

### Deferred consent and the parent-practitioner relationship

Practitioners were asked: ‘Do you think that approaching a parent/family member for deferred consent for their child’s participation in an emergency/urgent care trial can have a negative impact upon the parent/family member and practitioner relationship?’ Views were divided, whereas 44.4% (n = 20/45) did not believe there would be any negative impact, over half (n = 25/45, 55.5%) felt there could be either a small (defined as ‘A little’) (n = 20/45, 44.1%) or a fair amount of negative impact (n = 5/45, 11.1%). No practitioners indicated that approaching parents for deferred consent would have significant (‘A lot’) negative impact upon the parent-practitioner relationship.

There was a significant difference in views on the impact of deferred consent upon parent-practitioner relationship depending upon whether or not practitioners had experience of using deferred consent (p = 0.01). As shown in Table 2, a higher percentage of those who were experienced in deferred consent (n = 16/27, 59%) responded that the method would not negatively impact upon the parent-practitioner relationship, compared to those who had not previously used deferred consent (n = 4/18, 22%).

**Table 2:** **Do you think that approaching a parent/family member for deferred consent for their child’s participation in an emergency/urgent care trial can have a negative impact upon the parent/family member and practitioner relationship? (n** = **45)**

	**No (Not at all)**	**Yes (a little, a fair amount or a lot)**	**p-value**
Experience in deferred consent	16 (59)	11 (41)	0.01
No experience in deferred consent	4 (22)	14 (78)	
Consultant	9 (56)	7 (43)	0.24
Research nurse	11 (38)	18 (62)	
Involved in administering interventions	13 (50)	13 (50)	0.38
Not involved in administering interventions	7 (37)	12 (63)	

Figures are n (%), percentages are rounded to one decimal place.

There was no significant association between whether a practitioner was involved in administering interventions that were part of an EC clinical trial (p = 0.4) and perceived impact of deferred consent on the parent-practitioner relationship. Although not statistically significant (p = 0.2) a higher percentage of nurses (n = 18/29, 62%) stated that asking a parent for consent in EC could have a negative impact upon the relationship with parents and families when compared to consultants (n = 7/16, 43%).

In their free text responses experienced practitioners reflected on how families were generally happy to be approached after their child had been randomised: *“Nearly all families were openly receptive and wanted their child to be involved”* (P1, experienced in deferred consent) or indicated how parental responses to deferred consent in the emergency care setting had not been problematic: *“I have found that gaining deferred consent has been straightforward overall”* (P16, experienced in deferred consent).

### A tailored approach to recruitment

The emergency setting posed difficulties for clinical trial recruitment. Nine practitioners (20%) from four trials stated that they had deviated from the consent model outlined in the trial protocol. Additional comments at end of the questionnaire suggested that deviations occurred when parents had not been present when a child was receiving emergency treatment and therefore practitioners had been unable to provide brief information about the trial to parents. Practitioners commented on how parents were highly variable in their willingness to engage in a recruitment discussion and make a decision about trial entry when children are critically ill. Therefore practitioners needed to gauge the preferences of individual families and tailor their approach accordingly:

*“Some families can only think about and focus on their child getting better. They don't want to have to read, talk, as well as make a decision about research during this time. However some families do and are positive about research it’s a mix of people with different views and you approach them and see how they respond. If it’s negative, give them more time”* (P27, experienced in deferred consent).

Practitioners emphasised importance of appropriate timing when initially approaching parents about a trial. They wrote about how the deferred consent method had enabled research nurses to approach parents at a time when they were better able to process information, in comparison to approaching them when in the crisis of emergency treatment: *“When seeking deferred consent from parents we are first of all doing it at a time when we believe the parents can take in and process additional information in a rational and reasoned manner”* (P17, experienced in deferred consent).

### Recruitment and communication

Regardless of their experience of deferred consent, some practitioners described how clinical trial recruitment could strengthen relationships with patients, “*Opens up communication further*” (P14, not experience in deferred consent), mainly by helping to enhance communication between parents and practitioners about research, *“Often the parents feel they are contributing to research in the area which aids to conversation and communication generally between research nurse, clinical staff and the family”* (P36, experienced in deferred consent), or their child’s condition and treatment: *“It offers a chance for more in depth discussion about the baby’s condition”* (P25, experienced in deferred consent).

Clarity of communication was viewed as important when broaching a clinical trial that involved deferred consent. Practitioners wrote of how it was important to clearly explain to parents why deferred consent was being used: *“As long as the person introducing the trial and taking consent is clear about why deferred consent is being used, all the parents I have spoken to have had no issues”* (P10, experienced in deferred consent) as well as to clarify that any decisions parents made about clinical trial entry would not impact upon the clinical care of their child*: “It is made clear that not taking part in the study has NO bearing on their subsequent management in the trials where informed consent required prior to administration of trial substance”* (P42, experienced in deferred consent).

### The appropriateness of deferred consent

Many of those who were experienced in deferred consent reflected upon the method in the free comments box. Some described how they felt that deferred consent could be useful in clinical trial recruitment: *“The use of deferred consent within the emergency care setting is a valuable tool, which should be considered more often”* (P10, experienced in deferred consent). Others described how they felt the method was ethically sound: *“I believe there are absolutely no ethical issues with the framework that is in place”* (P45, experienced in deferred consent), or how families had no concerns about recruitment taking place after the intervention: *“No real concerns about the fact that the intervention has already been done”* (P29, experienced in deferred consent).

In contrast, one practitioner described how some parents felt that their voluntariness had been compromised by their child’s entry into a trial without their prior consent: *“Some parents felt that the decision had been taken out of their hands by the trial line (*central venous catheter) *already being inserted”* (P30, experienced in deferred consent). Another clinician seemed to reflect positively on how parents were unable to distinguish between research and standard care. This practitioner appeared to focus primarily on how deferred consent improved recruitment rates rather than its impact on the quality of recruitment: *“Deferred consent if appropriate for that child/trial is really improves recruitment to the clinical trials as the procedure has already occurred and doesn’t seem as much as a deviation from standard care to most families”* (P36, experienced in deferred consent).

### Training and guidance

Although all practitioners sampled were experienced in clinical trial recruitment, just over half (n = 25, 55.6%) felt they would benefit from training to assist them in discussing emergency/urgent care trials with families and obtaining consent. Their suggestions included: specialised training courses or workshops (n = 3); structured guidelines (n = 3), peer support, such as shadowing experienced trial recruiters or an online forum (n = 3), reading applicable research findings (n = 1), or openly accessible DVD or online materials (n = 1). The need for guidance on seeking deferred consent with bereaved parents was emphasised by two practitioners, both of whom emphasised the complexities of approaching bereaved parents for consent and the need for research to inform future practice:

*“There needs to be a discussion on seeking deferred consent from bereaved parents. When is the best time to approach, where, by whom, how should it be done, is it actually necessary?…does approaching these parents cause added stress/upset/confusion/-knowing how these parents feel would be very interesting indeed. This really needs to be debated by those involved in paediatric research”* (P17 experienced in deferred consent).

*“Different options in the protocol for consent that allows the practitioner to use their clinical judgement. E.g. when a patient dies before the consent is signed and although you have already approached the family prior to this you feel that it would be inappropriate to force them to make a decision when their child has just died. If following the protocol you would need to get an answer ideally within 48 hrs of randomisation. This could have an extremely negative impact on the family and influence their thoughts about participating in research in the future”* (P18 experienced in deferred consent).

## Discussion

Deferred consent provides a means of addressing the practical and ethical difficulties of obtaining prospective consent in an emergency situation, thus enabling medical research in children to precede that may benefit society as a whole. Practitioners’ views on deferred consent differed depending upon whether or not they were experienced in the deferred consent method. Negative perceptions were held by those who had not used deferred consent, particularly when responding to questions about the impact of clinical trial recruitment upon the parent-practitioner relationship. Those who had not used deferred consent described how parents can have a negative perception of EC clinical trials, which impacted upon the parent-practitioner relationship by making practitioners feel uncomfortable in interactions that took place after approaching parents about a trial [7]. In contrast, those who had used deferred consent described the positive impact that EC clinical trial recruitment can have upon the parent-practitioner relationship. Such findings underline the need to incorporate the views of practitioners with first hand experience of deferred consent should be considered in the design and ethical review of future paediatric EC trials; the design and ethical review of such trials should not solely be informed by the beliefs of those without experience of using deferred consent. These findings also highlight the importance of conducting empirical research to inform debate around key ethical problems [38,41].

Our findings support previous literature which outlines the practical and ethical complexities of seeking consent in the EC setting [4,6]. Practitioners perceived trial recruitment as an additional burden upon parents who were already anxious about their sick child [7,15,42]. Some questioned the ability of parents to process trial information when they arrived at hospital; particularly practitioners who had not used deferred consent. These practitioners raised concerns about approaching families in an emergency situation and reported that recruitment was a potential burden for families who were situationally incapacitated [7,11]. Such practitioners described how consent prior to randomisation could not be ‘fully’ informed as parents lacked capacity to digest trial information and make an informed decision about their child’s participation in a trial. These accounts contrasted with to those who had used deferred consent, who stated that this method enabled them to approach families when they were able to ‘take in and process’ trial information. Findings from practitioners with experience of deferred consent suggest that this consent method assists capacity to make informed decision making in an emergency situation in ways that the conventional approach of seeking of prior consent cannot. Moreover, our findings raise questions about the purpose of prior consent for EC trials as practitioners’ accounts indicate that this method cannot satisfy ethical and clinical practice guidelines on informed consent [6,24,30]. If parents are unable to digest information about the nature, significance, implications and risks of the study and make informed autonomous decisions, then is it inappropriate to be placing such demands upon them? Indeed, if they unable to process trial information then prior consent becomes only an administrative exercise to protect the legal interests and obligations of the health provider rather than respecting key bioethical principals such as respect for autonomy. The ethical validity and feasibility of obtaining informed consent or even providing brief information as part of a continuous consent approach is therefore in doubt [11].

The importance of timing and experiential learning in determining when is appropriate for practitioners to broach a trial in emergency care settings was evident in our findings. By deferring consent practitioners were able to provide time for parents to consider the trial information and have the opportunity to ask questions, as well as allowing practitioners to use their judgement to gauge when was the best time to make the approach [8]. A quarter of practitioners reported having to modify the consent model defined in the trial protocol because they were unable to provide brief prospective information. This reflects the often complex and time constrained emergency care setting [4]. Parents were not always available when a child was admitted, or there was insufficient time to provide brief information prior to randomisation. Brief information provision as part of a staged approach to consent may therefore not be practicable in the paediatric emergency care setting. Such observations are consistent with literature which has shown how the multifaceted nature of clinical practice is not always reflected in trial protocols, creating further difficulties in seeking consent [43].

Deferred consent was valued by those experienced in the approach. First-hand experience with deferred consent appeared to dispel some of the anxieties about the method. Indeed, almost all of those with experience of the method supported the use of deferred consent [44] and highlighted how it had enabled them to defer approaching parents about a trial to a time which was more ethically appropriate, when parents were less stressed and had improved capacity for informed decision making [11]. Such findings suggest that if conducted sensitively, and in a time appropriate manner, the deferred method can assist, rather than impede, parental decision making. However, a concern raised by one practitioner echoes that of some critics who state that voluntariness has already been compromised to some extent by the intervention being administered prior to recruitment [45]. Our findings raise questions about whether deferred consent limits parental autonomy as the intervention had already occurred [46]. The deferred consent approach may result in trial information being provided at a more appropriate time, when parents are more able to process and provide informed decisions’, yet what parents are consenting for has essentially been restricted. After all, deferred consent in this situation is about permission to continue in the study, or for use of data obtained, rather than to take part in the study [2,11,30]. Critics have argued that if parents are present at the time of recruitment and brief information is not provided, then deferred consent compromises individual autonomy and does not comply with the clinical practice guidelines on informed consent [11,24,30].

A further ethical issue was evident in comments made by a practitioner who expressed the value of deferring consent in terms of accrual rather than quality of recruitment. Although high accrual to paediatric clinical trials is important to inform future clinical practice, recruitment rates should not take precedence over ethical recruitment conduct [24,47]. Further research is required on communication between practitioners and parents in trial recruitment discussions to explore question such as: how do practitioners describe deferred consent to parents? And do they explain how a trial is different from routine clinical practice? [48].

Practitioners’ responses could help to inform training and guidance to inform the ethical conduct of this approach to seeking consent for UK clinical trials in emergency and urgent care settings. Considered alongside the wider literature, our study findings suggest this training would benefit from a focus on the process of communicating with families about a trial, the difference between standard care and clinical trials, and the importance of timing when approaching parents in this challenging setting [29,48]. Training would also benefit from consideration of which bioethical principles should inform recruitment practice. Research is also necessary to explore the views of parents who have been approached about EC trials to see if practitioners’ perceptions and reports of families’ responses to the different consent methods are echoed by families themselves.

### Limitations and future research

Deferred consent was used in only one of the 13 trials reported by this sample, which is likely to be due to the relatively recent changes in UK legislation (2008) to develop protocols using this method and the sample containing trials from 2004 onwards [27]. However, approximately half the study sample were experienced in deferred consent and half were not, providing an opportunity to compare and contrast views. Our study reports on a structured online survey, which limits the types of insights that it can provide. Moreover, we only report on the views of practitioners. The views of parents and children may be very different to those of practitioners, and as we note above, further work is needed to investigate the views of family members who have been on the receiving end of deferred consent. In-depth qualitative data will be collected in the next stages of the CONNECT study to explore the views and opinions of all stakeholders in CATCH. As well as interviews with recruiting practitioners, this will involve interviews with children and parents, including bereaved parents, in order to draw normative conclusions [49]. This study was not powered to detect differences between the groups. Due to the small sample size it is important that absence of statistical significance is not interpreted as no difference between the groups compared. Our sample was also non random and we were unable to report response rates so our findings should be interpreted with caution.

While the use of purposive sampling helped to access the views of practitioners who were experienced in deferred consent as well as those without such experience, it should be noted that all sampled practitioners with experience of deferred consent were from the CATCH trial. This is a low risk medical device trial [35]. Further research is required to explore clinician experiences in differing trial types (e.g. drug trials, and trials comparing less widely used treatments), if the deferred consent method is to be utilised more widely in the future. Research is also required to explore whether practitioners’ ethical orientations impact on their views on the acceptability and use of deferred consent in this setting.

Findings suggest that future protocols for clinical trial recruitment in emergency care should acknowledge the ethical implications of broaching a trial in urgent or emergency care and outline how parents should only be approached when it is judged by the practitioner as being ethically viable to do so [50]. One solution may be to outline options within the trial protocol to acknowledge practitioner’s personal judgement and provide flexibility to protect families in this setting. This could include options such as stages of consent, including brief information provision prior to randomisation if the practitioner deems it to be appropriate. Further research is required to explore this proposal. Research is also required to establish whether and how bereaved parents ought to be approached about a clinical trial. Such research should focus on how consent processes could avoid distressing such a vulnerable group and provide recommendations for future recruitment practice [19,51].

## Conclusions

The highly emotive and time limited emergency care setting compromises parental capacity to provide informed consent for their child’s participation in a clinical trial. The views of practitioners experienced in seeking deferred consent suggest that if conducted sensitively, and in a time appropriate manor, deferring consent can assist rather than impede autonomous decision making. However, evidence on the views of children and parents, including parents whose child has died after an emergency admission, are vital to draw normative conclusions. Our findings highlight how the views of practitioners with first hand experience of deferred consent should be incorporated into the design and ethical review of future paediatric EC trials. Further research with parents and practitioners is required to inform training and normative guidance to assist the ethical conduct of this relatively new approach to seeking consent for UK clinical trials in emergency and urgent care settings.

## Competing interests

The authors declare that they have no competing interests.

## Authors’ contributions

KW, LF, CG and BY conceived and designed the experiments. KW performed the experiments. KW, LF, CG and BY analyzed the data. KW, LF, CG and BY contributed to the design of materials and analysis tools. KW, LF, CG and BY wrote the paper. All authors read and approved the final manuscript.

## Pre-publication history

The pre-publication history for this paper can be accessed here:

http://www.biomedcentral.com/1472-6939/14/45/prepub

## Supplementary Material

Additional file 1 Recruiting Practitioner Survey: Connect Study. Click here for file
