# Supplementary material for: How experience makes a difference: practitioners’ views on the use of deferred consent in paediatric and neonatal emergency care trials
Source: BMC Med Ethics. 2013 Nov 6;14:45. doi: 10.1186/1472-6939-14-45 (PMC4228267; doi:10.1186/1472-6939-14-45)
Supplement: Additional file 1 — Recruiting Practitioner Survey: Connect Study. [file 1472-6939-14-45-S1.docx]

**Additional file 1**

RECRUITING PRACTITIONER SURVEY: CONNECT Study

The aim of this survey is to establish the types of consent methods used in UK emergency and urgent care paediatric clinical trials, the number of trials conducted since 2004 (Medicines for Human Use (Clinical Trials) regulations), as well as who is involved and any training and support needs. This includes trials conducted with neonates.

Please complete this survey if you are someone involved in the recruitment of families to paediatric emergency or urgent care trials. This includes practitioners who have experience of discussing trials with families even if they've not been the ones seeking consent.

PLEASE COMPLETE ONE QUESTIONNAIRE FOR EACH TRIAL YOU HAVE BEEN INVOLVED IN. ONLY COMPLETE PART B ONCE AS THESE ARE NOT TRIAL SPECIFIC QUESTIONS. We have kept the questionnaire very short for this reason. If you don't have time to complete more than one, please complete one questionnaire about the trial you have most recently been involved in.

Please answer the following questions by ticking your selected answer, or writing in the space provided. When complete, press the 'Done' button to submit your answers. Many thanks for taking the time to complete this survey.

* = answer that requires an answer

**PART A**

1. Number of paediatric emergency and urgent care trials you have been involved in since 2004:

2. Your Job title:

3. Name of the Trial (to which this questionnaire relates):

4. Age range of children eligible for inclusion:

5. Number of UK trial sites:

6. Trial start and end date (if known):

7. Were you directly involved in delivering a medical intervention as part of this trial?: (Yes, if yes, go to question 8/No, if no, go to question 7.b below):

7.b IF NO could you please describe your role in relation to the trial (e.g. research nurse responsible for trial recruitment):

8. What form of consent/assent was used in the trial? (Please tick all that apply): a. Informed prospective assent (from child before enrolment in trial)/ b. Deferred assent (from child after enrolment in trial)/c. Informed proxy prospective consent (from parent/guardian before enrolment in trial)/d. Deferred proxy consent (from parent/guardian after enrolment in trial)/e. Continuous proxy consent (stages of consent e.g. exchange of preliminary information followed by informed consent when appropriate)/ f. Other (please specify)

9. If you have used continuous consent (e.g. stages of consent) please describe below the types of consent obtained and when they were obtained (e.g. abbreviated written and verbal information before medical intervention followed by detailed written and verbal information afterwards):

10. If you use have used deferred proxy consent, please state in the box below the ideal time frame you would intend to use for deferred proxy consent in this trial (e.g. within 24hours post trial enrolment):

11. Have you ever had to deviate from the consent model outlined in the trial protocol? (Yes/No)

12. What was the written information provided to parents? (Please tick all appropriate answers below): a. Full patient information sheet (detailed participant information form)/ b. Abbreviated information (short participant information form)/ c. Abbreviated information upon enrolment followed by all-embracing information when appropriate/ d. Posters/e. Other (please specify)

13. Were 'child friendly' information materials provided by the trial team to assist the informed assent process? (Yes/No/Not applicable)

14. Were children or family members involved in the development of the recruitment or consent/assent materials? (Yes/No/Not sure)

**PART B (Only complete once)**

15. Generally, how do parents react when a trial is initially mentioned or broached in a paediatric emergency/urgent care situation? (Positively/somewhat positively/ Mixed proportions of positive/ Negatively/somewhat negatively and negative/ Uncertain)

16. How well do you feel parents/family members understand the trial information given to them in an emergency/urgent care situation? (Very well/well/unsure/Not well/Not at all well)

17. Do you think that approaching a parent/family member for deferred consent for their child’s participation in an emergency/urgent care trial can have a negative impact upon the parent/family member and practitioner relationship? (Not at all/ A little/ A fair amount/ A lot)

18. Please could you describe your experiences of the impact of recruitment upon parent/family member and practitioner relationship in this setting? (Even if you think there is little or no impact):

19. Do you feel you would benefit from any training to assist you in discussing emergency/urgent care trials with families and obtaining consent? (Yes/No/Not sure)

b. If yes, could you please describe below what type of training (or support) would benefit you:

20. If you have any further comments that you think would be useful to inform future recruitment practice in paediatric emergency/urgent care trials please state here:
